# Proline-Rich Hypervariable Region of Hepatitis E Virus: Arranging the Disorder

**DOI:** 10.3390/microorganisms8091417

**Published:** 2020-09-15

**Authors:** Milagros Muñoz-Chimeno, Alejandro Cenalmor, Maira Alejandra Garcia-Lugo, Marta Hernandez, David Rodriguez-Lazaro, Ana Avellon

**Affiliations:** 1 Hepatitis Unit, National Center of Microbiology, Carlos III Institute of Health, 28220 Madrid, Spain; milagrosmch@hotmail.com (M.M.-C.); alex.cenalmor8@gmail.com (A.C.); maleja.garcia.l@hotmail.es (M.A.G.-L.); 2 Laboratorio de Biología Molecular y Microbiología, Instituto Tecnológico Agrario de Castilla y León (ITACyL), 47071 Valladolid, Spain; hernandez.marta@gmail.com; 3 Microbiology Division, Faculty of Sciences, University of Burgos, 09001 Burgos, Spain; drlazaro@ubu.es; 4 CIBER Epidemiology and Public Health, 28029 Madrid, Spain

**Keywords:** hepatitis E virus, HEV, PPR, HVR, hypervariable, hyper-proline, IDR

## Abstract

The hepatitis E virus (HEV) hypervariable region (HVR) presents the highest divergence of the entire HEV genome. It is characteristically rich in proline, and so is also known as the “polyproline region” (PPR). HEV genotype 3 (HEV-3) exhibits different PPR lengths due to insertions, PPR and/or RNA-dependent RNA polymerase (RdRp) duplications and deletions. A total of 723 PPR-HEV sequences were analyzed, of which 137 HEV-3 sequences were obtained from clinical specimens (from acute and chronic infection) by Sanger sequencing. Eight swine stool/liver samples were also analyzed. N- and C-terminal fragments were confirmed as being conserved, but they harbored differences between genotypes and were not proline-plentiful regions. The genuine PPR is the intermediate region between them. HEV-3 PPR contains a higher percentage (30.4%) of prolines than other genotypes. We describe for the first time: (1) the specific placement of HEV-3 PPR rearrangements in sites 1 to 14 of the PPR, noting that duplications are more frequently attached to sites 11 and 12 (AAs 74–79 and 113–118, respectively); (2) the cadence of repetitions follows a circular-like pattern of blocks A to J, with F, G, H, and I being the most frequent; (3) a previously unreported insertion homologous to apolipoprotein C1; and (4) the increase in frequency of potential N-glycosylation sites and differences in AAs composition related to duplications.

## 1. Introduction

Hepatitis E virus (HEV) infection is an important component of enteric-transmitted liver diseases and has a significant impact on public health. The number of new HEV infections has increased in recent years in the industrialized countries of the European Union [1]. HEV genotype 3 infection (HEV-3) is a viral zoonosis transmitted to humans through consumption of meat from infected animals, mainly pig [2,3,4], wild boar [5,6,7], and deer [8]. HEV-3 is spreading worldwide and is the cause of acute mainly self-limited hepatitis. In immunocompetent and immunocompromised patients, hepatitis can be fulminant, while chronic infection has been only described in immunocompromised.

The HEV genome is a positive-sense non-enveloped single-stranded RNA molecule of 7.2 kb containing three partially overlapping open reading frames (ORF1, ORF2, and ORF3) [9]. ORF2 encodes the viral capsid protein and contains neutralizing epitopes of virus particles and is also the target of humoral immune response [10,11]. ORF3 protein is essential for virion secretion [12] and this protein has ion channel activity that is required for the release of infectious virus [13]. Seven putative domains have been identified in ORF1: methyltransferase (MTase), Y domain, putative papain-like cysteine protease (PCP), the proline-rich hinge domain (PPR) or hypervariable region (HVR), the X domain, putative RNA helicase, and RNA-dependent RNA polymerase (RdRp) [14].

HVR is located in the ORF1 proliprotein between the PCP and X domain. It is known as the “hypervariable region” because it is the one with the greatest divergence in the entire HEV genome [15]. As a consequence, it is difficult to obtain complete and satisfactory alignment between all the HEV genotypes. HVR is characteristically rich in the amino acid proline (in fact, the HVR and PPR domains overlap), for which reason it is also known as the “polyproline region” (PPR), and contributes to viral replication efficacy and adaptation [15,16,17].

HVR-PPR (PPR, hereafter) function is not fully understood, but in vivo and in vitro studies have shown that deletions in this region do not influence virus viability. Conversely, larger or nearly complete PPR deletions cause virus attenuation, suggesting that PPR is involved in viral pathogenesis [18]. A study of HEV replicons led to the suggestion that, although there is a degree of specificity by genotype, PPR may be functionally exchanged among them. In fact, sequence composition can modulate HEV RNA replication and infectivity [16]. A 3D model for predicting functional sites demonstrated that protein to protein interactions help regulate virus replication. This finding, along with the variation in length among genotypes, supports the hypothesis that PPR is also involved in host adaptation.

Originally, a 105-amino acid (AA) fragment was proposed as being a PPR [14]. When more sequences became available, it was observed that the first 35 AAs might not be included in the region and, therefore, it was concluded that the genotypes 1–4 had a PPR comprising AAs 70–72, 68, 80–86, and 84 [18]. PPR was subsequently found to feature conserved sequences (TLYTRTWS and RRLLXTYPDG) at the N- and C-terminal sides, respectively [15].

The PPR AA sequence is known to be different by 71% among genotypes, 31% within HEV-1, 41% within HEV-3, and 46% within HEV-4 [18]. The degree of sequence variability in HEV-1 is lower than in zoonotic HEV-3 and HEV-4, which may be related to their adaptation to a wide range of hosts [15].

The PPR length in HEV-3 was reported to be 107–172 AAs [19]. The sequences of the subtype that infect rabbits (HEV-3ra) have the shortest PPR and the sequences of subtype 3f can be divided into two groups: short (246 nucleotides [NT]) and long (333 NT)). However, subtypes 3b, 3c, and 3i have the same length (243 NT) and PPR from subtype 3e are 246 NT long [20,21]. The different length of the sequences is thought to be due to the presence of insertions. Analysis of isolates from samples of immunocompromised patients with chronic infection, revealed that this region can acquire insertions over time and that these fragments can arise either from the viral genome (duplications of PPR and RdRp, PPR + X-domain (20)(20)(20) [20,22,23,24], or from human genes, which, until now, have been identified as the genes coding for human ribosomal proteins S19 and S17 [25,26], and those of human tyrosine aminotransferase (TAT), human inter-α-trypsin inhibitor (ITI), eukaryotic translation elongation factor (EEF1A1P13), the 18S ribosomal pseudogene (RNA 18SP5), a kinesin family member (KIF1B), and zinc finger protein (ZNF787) [24]. In vitro studies have suggested that human inserts may be related to the improvement in virus replicative capacity [25,26,27]. However, a 186-nucleotide insertion derived from PPR and RdRp also allows HEV adaptation in A549 cell line [23].

The aim of this study was to analyze the HEV virus PPR in different genotypes in an attempt to make sense of the apparent disorder. To this end, we examined the genome variability, sequence length, location, and potential implications of genomic rearrangements in each genotype and subtype.

## 2. Materials and Methods

### 2.1. Newly Obtained Sequences

We obtained 137 new PPR sequences from human samples for this study, 126 of which were from RNA extracts of patients with acute HEV infection, and 11 were from the follow-up of three chronic patients: CR1 (*n* = 5, HEV-3c), CR2 (*n* = 3, HEV-3f) and CR3 (*n* = 3, HEV-3f). Eight swine stool/liver samples (*n* = 1 HEV-3c and *n* = 7 HEV-3f) were also included in the analysis.

RNA was extracted with the Magna Pure L.C. © System (Roche Diagnostics, Mannheim, Germany) automatic extraction from 200 µL of serum samples. After RNA extraction, complementary DNA was transcribed with a Transcription First Strand cDNA Synthesis kit (Roche Diagnostics, Mannheim, Germany) using random hexamers and 20 µL of cDNA were obtained from 10 µL of RNA extract, following the manufacturer’s recommendations. PPR fragments were obtained through nested PCR, as previously described [19]. Afterwards, amplification products were purified with Illustra ExoProStar 1-step (VWR International Eurolab S.L., Radnor, PA, USA) and sense and antisense DNA strands were both sequenced by the Sanger method. GenBank accession numbers are MT899272 to MT899416.

### 2.2. Genbank Sequences

Initially, 49 reference sequences proposed by Smith et al. were included [28]. Additionally, sequences whose GenBank accession numbers are available as supplementary material 1 corresponding to HEV-1 (*n* = 61), HEV-3 (*n* = 369), HEV-4 (*n* = 95), and HEV-8 (*n* = 4) were analyzed.

### 2.3. Sequence Analysis

#### 2.3.1. Total Number of Sequences Included

HEV-1 (*n* = 70), HEV-2 (*n* = 3), HEV-3 (*n* = 533), HEV-4 (*n* = 106), HEV-5 (*n* = 1), HEV-6 (*n* = 2), HEV-7 (*n* = 2), and HEV-8 (*n* = 6).

#### 2.3.2. Consensus Definition

PPR consensus by genotype was obtained from reference and study sequences as follows: the AA consensus sequence was established according to the most frequent AA in each position (aligned with the MegAlign program; DNASTAR, Lasergene Inc., 12.3.1 Madison, WI, USA).

#### 2.3.3. Limits of PPR

Due to the great variability of this region, we analyzed PPR in fragments, choosing histidine (H) as the starting position and aspartic acid (D) as the final position. Three fragments were examined: the initial 32 AAs in the N-terminal region, the intermediate region of variable length, and the final 12 AAs of the C-terminal region.

#### 2.3.4. Amino Acid Composition

AA composition of each PPR sequence was calculated with the EMBOSS Peptats program, available at https://www.ebi.ac.uk/Tools/seqstats/emboss_pepstats/.

#### 2.3.5. Residue Variability

This was calculated as the percentage of discordant AAs with respect to the consensus AA of each genotype. The average residue variability of each fragment was also determined.

#### 2.3.6. Sequence Homology Analysis

This was calculated as the percentage of conserved AAs with respect to the consensus AA of each genotype.

#### 2.3.7. Analysis of Insertions

In study sequences, a BLAST (https://blast.ncbi.nlm.nih.gov/Blast.cgi) was performed to determine the origin of insertions.

#### 2.3.8. Regulation Sites Analysis

Potential ubiquitination sites were analyzed using the BMD-PUB server (http://bdmpub.biocuckoo.org/prediction.php), with a threshold value of a >0.3 average potential score. Potential acetylation sites were identified using the PAIL server (http://bdmpail.biocuckoo.org/prediction.php) with a value of >0.2 of the average potential score. Potential phosphorylation sites were identified using the NetPhos 2.0 server (www.cbs.dtu.dk/services/NetPhos/) with a value of >0.5 of the average potential score. Finally, potential N-linked glycosylation sites were analyzed using the NetNGlyc 1.0 server (www.cbs.dtu.dk/services/NetNGlyc) with a value of >0.5 of the average potential score.

### 2.4. Statistical Analysis

Qualitative variables were analyzed with chi-square tests. Values of *p* < 0.05 were considered to be significant.

## 3. Results

Three genomic regions were differentiated: the PPR-N-terminal (genome region encompassing the first 32 AAs); the PPR-C-terminal including the final 12 AAs; and the PPR intermediate region. The PPR-N-terminal (Table 1) and PPR-C-terminal (Table 2) regions were relatively highly conserved among genotypes. By contrast, the length and variability among genotypes differed in the intermediate region (Table 3), where there are large differences in length due to insertions, duplications and deletions.

### 3.1. PPR-N-Terminal Region

The average residue variability of this region was 2.76% in HEV-3, 2.13% in HEV-4, and 1.34% in HEV-1, with no significant differences between them. Most genotypes had 31 AAs, although the HEV-2 PPR-N-terminal was the shortest (27 AAs), and HEV-1 was the longest (32 AAs), with an extra valine at position 23. Proline was present at the highly conserved positions 8 and 32, but the most common AA was serine, even though its percentage varied between genotypes, being higher in the zoonotic (21.7%) than in the non-zoonotic (13.8%) genotypes (*p* < 0.05).

### 3.2. PPR-C-Terminal Region

The average residue variability of this region was 1.14% in HEV-1, 0.72% in HEV-3, and 0.19% in HEV-4, with no significant differences between them. All genotypes had nine AAs, and residues 130–133 and 136–138 were conserved in all genotypes. Proline was present and conserved in position 136.

### 3.3. PPR Intermediate Region

#### 3.3.1. Variability and Composition

The average residue variability of this region was highest in zoonotic HEV-4 (28.20%) and HEV- 3 (24.20%) than in non-zoonotic HEV-1 (12.21%) (*p* < 0.05). Proline was present throughout the entire region, being the most common AA, with an average of 22.8%. The proline composition differed among the genotypes (25.41% in HEV-1, 19.24% in HEV-2, 30.43 in HEV-3, 25.42% in HEV- 4, 17.33% in HEV-5, 21.05% in HEV6, 22.73% in HEV-7, and 20.60% in HEV-8), the amount being significantly higher in HEV-3 (*p* < 0.05). Furthermore, the prolines in positions 117 and 121 were conserved in all genotypes, except for HEV-1. Arginine at position 128 was also highly conserved across genotypes. In addition, HEV-2, with only three available sequences, had a significantly higher percentage of glycine (16.04%) compared with the other genotypes (6.11%) (*p* < 0.05).

#### 3.3.2. Length, Deletions and Insertions

HEV-1 and HEV-2 were 60 and 62 AAs long. Consensus alignment of the two genotypes demonstrated that HEV-2 had seven more AAs in positions 36, 43–47, and 121, and five fewer AAs in positions 116 and 126–129 regarding to HEV-1. One motif (GHLDA43-47) was only present in HEV-2. We identified many differences between subtypes 2a and provisional 2b with 18 of 62 AAs conserved in both. In addition, one more AA was found in one of the two available HEV-2a sequences. The most recently described genotypes were HEV-5, HEV-6, HEV-7, and HEV-8, for which reason there are few available sequences, but we nevertheless observed some differences between them. The first difference was that the lengths were 66 AAs in HEV-7, 72 in HEV-8, 75 in HEV-5, and 76 in HEV-6. All of them exhibited many AA differences between their subtypes. HEV-6 had 35.5% sequence homology between 6 and 6a, with values of 37.9% in HEV-7 and 65.3% in HEV-8. Consensus alignment identified two motifs that were only present in HEV-8 at positions 58–60 (LLX) and 91–93 (XAH), similar to HEV-6 and HEV-7, which had seven or eight additional AAs at positions 101–108. In the case of HEV-4, different lengths were found in distinct subtypes: HEV-4 and HEV-4g (73 AAs); HEV-4a, HEV-4c, and HEV-4d (73–75 AAs); HEV-4b (70–73 AAs), HEV-4e, and HEV-4h (74 AAs); HEV-4f (72 AAs); and 4i (69–74 AAs).

HEV-3 featured insertions, duplications and deletions. As a consequence, the HEV-3 sequence length ranged from 57 to 165 AAs. Deletions were frequent throughout the HEV-3 and HEV-4 intermediate region. Most deletions were of only one AA, but HEV-3ra and HEV-3g were shorter (deletions detailed in Table S1). HEV-3ra was 59 AAs long, with deletions from positions 52, 82–99 and 112. HEV-3g comprised 63 AAs due to deletions at positions 38, 40–41, 50–52, 63, and 81–82). Finally, HEV-4i sequences had a 5-AA deletion at positions 72 to 76.

Insertions were more frequent at positions 50 to 111 at different sites. Except for one sequence that duplicated complete PPR, the duplications usually appeared at positions 74–79 and 113–118, these being duplicate fragments of AAs from position 67. There were 10 duplication blocks (Blocks A–J) (Table 4). Usually, the first fragment to appear duplicated was that immediately adjacent to the positions where the insertion occurred. For instance, block H was the starting block in duplications inserted at position 117. The only exception to this was in the 35 sequences corresponding to HEV-3a in which the inserted block was I in position 113 (Table 4). Duplicated blocks were located one after the other, mainly in alphabetical order (and thereby in the same order as in the wild type PPR), although one of the blocks was occasionally skipped, or the cycle started with previous blocks, which recovered the alphabetical order (e.g., HIJABCDE). The most commonly repeated blocks were F, G, H, and I. In fact, we found one sequence (KJ917717) in which blocks F, G, H, I, and J were repeated up to three times in total. The HIJFG duplication was found in 189 HEV-3f sequences.

The longest PPR duplication (KT591534) exhibited a complex rearrangement including a duplication of the entire intermediate region plus four AAs (RRLL) from the PPR-C-terminal region plus eight AAs (SLKGFWKK) from RdRp and ten AAs (TSGFSSDFSP) from the PPR-N-terminal region (site 1—Table 3). Other complex sequences included a PPR duplication and an additional insertion corresponding to the following: transpeptidase family protein 85% homologous (MF444086); four AAs (RRLL) from the PPR-C-terminal region (KJ917704, MN646690, MN646691, and KJ917717); 13 AAs from the substrate-binding domain (*P. fluorescens,* 85% identity) (KJ917704); and 45 AAs from synthase (*Actinobacteria bacterium*) (KJ917720) (site 12—Table 3 and Table 4).

HEV genome insertions other than PPR were HEV-RdRp fragments are illustrated in Table 3 and Table 4. In two sequences (KC618402 and KC618403) we found a 24-AA RdRp motif (LRGLTNVAQVCVDVVSRVCGVSPG).

Finally, we found HEV-3 inserts of ribosomal proteins 17S, 18S, 19S, and L6 (RPS17, RNA18S, RPS19, and RPL6, respectively) and human genes such as ring finger protein 19A (RNF19A), eukaryotic translation elongation factor 1a1 (EEF1a1P13), zinc finger protein (ZNF787), glycine aminotransferase (GATM), inter-alpha-trypsin-inhibitor heavy chain H2 (ITIH2), and kinesin-like protein 1B (KIF1B). We noted an insertion of five AAs in the HEV-3j sequence (STLPS motif) of unknown origin. In addition to that described in HEV-3, we identified a single AA insertion between positions 122 and 123 in HEV-4 that was present in sequences from HEV-4c, HEV-4a, and HEV-4d.

#### 3.3.3. Specific Analysis of Newly Obtained HEV-3 Sequences

Ninety HEV-3f sequences from patients with acute infection had a 29-AA duplication of blocks HIJFG (site 12—Table 3 and Table 4), identical to six of the seven HEV-3f sequences obtained from swine stool/liver samples.

Regarding the follow-up of chronic patients, HEV-3f CR-3 patient had a 28-AA insertion (site 3—Table 3) related to human apolipoprotein C1 (100% identity) that was maintained in the three follow-up samples over one year. This human insert conferred an increase in the number of potential regulation sites: four acetylation, three ubiquitination, and five phosphorylation sites (4 serine and 1 threonine). In the case of the HEV-3c CR-1 patient, the first sequence had duplicated blocks (site 12—Table 3 and Table 4), but the duplication was lost in the subsequent four follow-up samples. The three HEV-3f sequences of CR-2 patient had no insertions.

The alterations of the number of potential regulation sites in the 91 sequences with duplication of blocks HIJFG (90 HEV-3f acute cases and one of HEV-3c CR-1 chronic infection) are as follows: Ubiquitination-suitable sites often increased by one site (range, 0 to 2); acetylation-suitable sites often increased by one site (range, −1 to 2); and potential phosphorylation sites often increased by six sites (range, 1 to 9) mainly due to the presence of serine. Regarding N-glycosylation 16 out of 91 (17.6%) sequences with duplication had at least one potential N-glycosylation site; on the contrary none of the sequences without duplications or the sequences with insertions had potential N-glycosylation sites (*p* < 0.05).

Table 5 compares the characteristics of AA composition of sequences with insertions, duplications and without either. We observed an increase in positively charged and a decrease in hydrophobic and aromatic AAs in sequences with human fragment insertion; and an increase of negatively charged and hydrophobic while polar and a decrease in aromatic AAs in sequences with duplications.

## 4. Discussion

The PPR-C-terminal and PPR-N-terminal regions cannot be considered truly hypervariable or hyper-proline regions. Although a 105-AA fragment was originally considered to be a PPR [14], this study confirmed that the disorder does not actually encompass the entire hypervariable region, and implies that the true PPR would be located between positions 33 and 129, as was previously suggested [18,29]. A high conservation rate of these fragments was observed intra-genotypically, but with specific inter-genotypic discrepancies (AA 2 and AA 29 in the N-terminal region). Furthermore, AA 30 allowed zoonotic and non-zoonotic genotypes to be differentiated. The high degree of conservation of the two zones flanking the PPR-intermediate region suggests that a possible function can be assigned to these zones, although this would require additional functional studies. Proline is not common in these terminal regions, the PPR-N-terminal region being particularly rich in serine, which might be crucial for protein phosphatases that control many cell functions [30].

The true PPR hypervariable region is thus the intermediate region flanked by the PPR-N- and PPR-C-terminal regions. HEV-1, 5, 6, 7, and 8 maintain their length, harboring 60, 75, 76, 66, and 72 AAs, respectively. These differences seem to be related to previously undescribed insertions as the sequence GHLDA in HEV-2. Previous studies reported high sequence similarity in HEV-1 [29]. There are few sequences available for the cases of HEV-2, 5, 6, 7, and 8, which makes it difficult to draw conclusions, but our study nevertheless revealed considerable diversity among the small number of available sequences of each genotype. This means that although phylogenetic studies usually exclude this region because of its high degree of divergence, its phylogenetic use might be suitable (Figure S1), especially when complemented by the analysis of other genome regions [15]. There is more proline in HEV-3 than in the other genotypes.

By contrast, the main zoonotic genotypes, HEV-3 and HEV-4, showed substantive differences in length due to insertions and deletions. Although this phenomenon has been previously reported [20,24,26,27], we describe for the first time the specific location of HEV-3 PPR rearrangements, noting that PPR duplications were more attached to specific locations (AAs 74–79 and 113–118). In this study we analyzed 723 HEV sequences, including 137 newly obtained sequences through Sanger sequencing. Next-generation sequencing may help researchers obtain hundreds of full genomes, but may give incorrect results in PPR when extreme rearranged sequences are assembled by mapping with reference genomes; thus, in these cases, it would be better to use de novo assembly or Sanger sequencing to obtain more reliable results [31]. Duplications affect HEV-3a, 3c, 3e, and 3f, and are described in acute and chronic infections. Additionally, a more thorough analysis of the duplications in HEV-3 show the previously unreported cadence of repetitions, which follows a circular-like pattern of blocks. Previous studies reported that HEV-3f was divided into HEV-3f-short and HEV- 3f-long [32], based on the specific duplication of blocks of HIJFG. Here we describe the same duplication in one HEV-3c sequence from a chronic patient who presented this duplication in their first sequence but not in the subsequent four follow-up samples.

In the sequences newly obtained for this study, duplications increased the frequencies of potential ubiquitination, acetylation, and phosphorylation sites, as described previously [24]. However, a previously unreported increase in the number of potential N-glycosylation sites was also observed. Considering the parallels with other viruses, similar rearrangements have been described in the JC polyomavirus, whose noncoding control region, related to replication and transcription, features a genomic rearrangement that increases the replication rate and viral gene expression in patients with progressive multifocal leukoencephalopathy [33,34]. Something similar occurs in cytomegalovirus cell-adapted strains that contain genomic arrangements located, in this case, in non-essential genes [35]. More similar to HEV PPR, the regulatory Nsp2 protein of porcine reproductive and respiratory syndrome virus (PRRSV) contains a highly conserved N-terminal enzyme domain, a highly conserved C-terminal transmembrane region, and a hypervariable intermediate region with differences in length between the European and North American strains [36]. The introduction of duplications in a highly conserved 3’-noncoding region of the Japanese encephalitis virus (JEV) was found to lead to increases in the production of RNA and of virus yield [37]. In respiratory syncytial virus, a duplication of 23 amino acids was observed in the C-terminal region of the attachment glycoprotein that resulted in the repetition of seven potential o-glycosylation sites. Such changes may influence the pathogenicity of the virus [38]. In duplications we described an increase of negatively charged AAs by contrast with those previously described [24]. Insertions from other HEV ORF1 proteins, such as RdRp, that do not correspond to any functional motif, or that increase the number of potential functional sites, have been found less frequently [24].

Apart from duplications, exogenous inserts, all of human origin, were located along the PPR affecting HEV-3a, 3c, 3f, 3h, and 3m. We describe a new inserted fragment (homologous to apolipoprotein C1) in three samples from a chronic patient. Apolipoprotein C1 in hepatitis C virus is related to morphogenesis and virus infection [39]. Furthermore, the insertion significantly increased the number of potential acetylation, phosphorylation, and ubiquitination sites that might be involved in host adaptation [15]. Cell culture studies have demonstrated a replicative improvement of HEV harboring PPR insertions of the inter-alpha-trypsin-inhibitor heavy chain H2 [20], or the S17 and S19 ribosomal genes [23], that gives rise to new ubiquitination, acetylation, or phosphorylation sites. It seems significant that although a wide range of animals are susceptible to HEV-3, the exogenous inserts described are all of human origin. Human fragment insertions increase the frequency of positively charged AAs as described before [24] and decrease hydrophobic and aromatic AA fractions.

Two of the three patients with a chronic infection in our panel presented PPR insertions, although this is a short number of patients, the frequency of rearrangements in chronic infected patients seem to be high in contrast to the findings of Lhomme et al. [20], who reported that, in the majority of chronic patients, the PPR did not show insertions during follow-up.

In contrast to the HIV and HCV hypervariable regions, in which HVR are related to the structural proteins that the host response forces to mutate, allowing the virus to evade neutralizing antibodies [40,41], HEV PPR as Rubivirus PPR, is considered an intrinsically disordered region (IDR), i.e., a protein domain that does not adopt a compact three-dimensional structure [15]. IDRs are a consequence of the viral interaction with hosts in a wide variety of host viruses, such as herpes simplex [42]. More structural studies are required to see how duplications affect protein conformation.

Independently of the significance of the insertions, their abundance and variety suggest that PPR is a region that tolerates the insertions well, without apparently affecting virus viability. Potential insertion sites in HEV ORF1 were identified by the combined use of transposon-mediated random insertion and selection in a subgenomic replicon system, but insertions in functional domains (Mtase, helicase, and RdRp) were not viable. However, immunofluorescence, immunoblot analysis, and luciferase activity measurement demonstrated that PPR insertions do not affect virus infectivity and facilitate viral production [43]. This may be of interest in genetic engineering and will require additional studies to determine what insert capacity PPR allows and its potential use.

## 5. Conclusions

We propose that the true proline-plentiful hypervariable region is flanked by the PPR-N- and PPR-C-terminal regions which while conserved, harbor differences between genotypes and are not proline-plentiful regions.

- We describe PPR length differences between HEV genotypes.
- We describe for the first time the specific location of HEV-3 PPR rearrangements in sites 1 to 14 of the PPR, noting that duplications are more attached to sites 11 and 12 (AAs 74–79 and 113–118, respectively). The cadence of repetitions follows a circular-like pattern of blocks A to J, with blocks F, G, H, and I being the most frequent. Duplicated fragments increase the frequency of potential N-glycosylation sites and negatively charged AAs.
- We identify a previously unreported insertion homologous to apolipoprotein C1 in a chronic patient sample.

## Figures and Tables

**Table 1:** HVR N-terminal region consensus amino acid from HEV-1 to HEV-8. Table shows consensus amino acid sequence for each genotype. AA variability: black, <15%; grey, 15–40%; white > 40%. Prolines in red. * Several HEV-3 sequences were shorter. Missing amino acids were not included in the calculation of the variability.

	1	2	3	4	5	6	7	8	9	10	11	12	13	14	15	16	17	18	19	20	21	22	23	24	25	26	27	28	29	30	31	32
HEV-1	**H**	**V**	**W**	**E**	**S**	**A**	**N**	**P**	**F**	**C**	**G**	**E**	**S**	**T**	**L**	**Y**	**T**	**R**	**T**	**W**	**S**	**E**	**V**	**D**	**A**	**V**	**S**	**S**	**P**	**A**	**R**	**P**
HEV-2	**H**	**E**	**W**	**R**	**S**	**A**	**N**	**P**	**F**	**C**	**G**	**E**	**S**	**T**	**L**	**Y**	**T**	**R**	**T**	**W**	**S**	**T**	**-**	**I**	**T**	**-**	**-**	**-**	**-**	**D**	**T**	**P**
HEV-3 *	**H**	**L**	**W**	**E**	**S**	**A**	**N**	**P**	**F**	**C**	**G**	**E**	**S**	**T**	**L**	**Y**	**T**	**R**	**T**	**W**	**S**	**T**	**-**	**S**	**G**	**F**	**S**	**S**	**C**	**F**	**S**	**P**
HEV-4	**H**	**S**	**W**	**E**	**S**	**A**	**N**	**P**	**F**	**C**	**G**	**E**	**S**	**T**	**L**	**Y**	**T**	**R**	**T**	**W**	**S**	**V**	**-**	**S**	**G**	**F**	**S**	**S**	**C**	**F**	**S**	**P**
HEV-5	**H**	**K**	**W**	**E**	**S**	**A**	**N**	**P**	**F**	**C**	**G**	**E**	**S**	**T**	**L**	**Y**	**T**	**R**	**T**	**W**	**S**	**T**	**-**	**S**	**G**	**F**	**S**	**S**	**N**	**F**	**S**	**P**
HEV-6	**H**	**K**	**W**	**E**	**S**	**A**	**N**	**P**	**F**	**C**	**G**	**E**	**S**	**T**	**L**	**Y**	**T**	**R**	**T**	**W**	**S**	**T**	**-**	**S**	**G**	**F**	**S**	**S**	**S**	**F**	**S**	**P**
HEV-7	**H**	**I**	**W**	**D**	**S**	**A**	**N**	**P**	**F**	**C**	**G**	**E**	**S**	**T**	**L**	**Y**	**T**	**R**	**T**	**W**	**S**	**V**	**-**	**S**	**G**	**F**	**S**	**S**	**D**	**F**	**A**	**P**
HEV-8	**H**	**V**	**W**	**D**	**S**	**N**	**N**	**P**	**F**	**C**	**G**	**E**	**S**	**T**	**L**	**Y**	**T**	**R**	**T**	**W**	**S**	**T**	**-**	**S**	**G**	**F**	**S**	**S**	**N**	**F**	**S**	**P**

**Table 2:** HVR C-terminal region consensus amino acid from HEV-1 to HEV-8. Table shows consensus amino acid sequence for each genotype. AA variability: black, <15%; grey, 15–40%; white >40%. Prolines in red. * Several HEV-3 sequences were shorter. Missing amino acids were not included in the calculation of the variability.

	130	131	132	133	134	135	136	137	138
HEV-1	**R**	**R**	**L**	**L**	**F**	**T**	**Y**	**P**	**D**
HEV-2	**R**	**R**	**L**	**L**	**H**	**T**	**Y**	**P**	**D**
HEV-3 *	**R**	**R**	**L**	**L**	**Y**	**T**	**Y**	**P**	**D**
HEV-4	**R**	**R**	**L**	**L**	**H**	**T**	**Y**	**P**	**D**
HEV-5	**R**	**R**	**L**	**L**	**H**	**A**	**Y**	**P**	**D**
HEV-6	**R**	**R**	**L**	**L**	**H**	**T**	**Y**	**P**	**D**
HEV-7	**R**	**R**	**L**	**L**	**F**	**T**	**Y**	**P**	**D**
HEV-8	**R**	**R**	**L**	**L**	**H**	**V**	**Y**	**P**	**D**

**Table:** (**a**)

PRP^(1)^+RdRp (3f)													1AA (3c, 3-Unk)						ApoC1 (3f)					RPS17^(1)^ (3f)							RPL6^(1)^ (3m) RPS19^(1)^ (3a) RNF19A^(1)^ (3h)							RPS17^(1)^ (3a) PPR^(1)^+RdRp (3c)					PPR (3e, 3f) EEF1a1P13^(1)^						
		1															2			3				4										5								6					7		
	33	34	35	36	37	38	39	40	41	42	43	44	45	46	47	48	49	50	51	52	53	54	55	56	57	58	59	60	61	62	63	64	65	66	67	68	69	70	71	72	73	74	75	76	77	78	79	80	81
HEV-1					**D**	**L**	**G**	**F**	**M**	**S**						**E**	**P**	**S**	**I**	**P**	**S**	**R**	**A**	**A**	**T**				**P**	**T**	**P**	**A**	**A**	**P**				**L**	**P**	**P**	**P**	**A**	**P**	**D**	**P**			**S**	**P**
HEV-2				**L**	**T**	**V**	**G**	**L**	**I**	**S**	**G**	**H**	**L**	**D**	**A**	**A**	**P**	**H**	**S**	**G**	**G**	**P**	**P**	**A**	**T**				**A**	**T**	**G**	**P**	**A**	**V**				**G**	**S**	**S**	**D**	**S**	**P**	**D**	**P**			**D**	**P**
HEV-3			**P**	**E**	**A**	**A**	**Y**	**A**	**A**	**P**						**A**	**P**	**D**	**M**	**G**	**L**	**P**	**S**	**G**	**T**				**P**	**S**	**S**	**A**	**S**	**D**	**I**	**W**	**V**	**L**	**P**	**P**	**P**	**S**	**E**	**G**	**S**	**A**	**I**	**D**	**P**
HEV-4	**L**	**E**	**P**	**C**	**A**	**P**	**D**	**L**	**P**	**P**						**P**	**V**	**E**	**T**	**D**	**T**	**P**	**V**	**A**	**V**				**D**	**V**	**P**	**P**	**P**	**A**	**T**	**S**	**A**	**Q**	**P**	**Q**	**P**	**P**	**A**	**P**	**E**	**R**	**A**	**A**	**P**
HEV-5	**F**	**E**	**T**	**G**	**A**	**A**	**D**	**Q**	**P**	**P**						**G**	**V**	**G**	**A**	**V**	**V**	**L**	**S**	**A**	**E**				**A**	**A**	**R**	**P**	**P**	**V**	**V**	**T**	**L**	**P**	**P**	**A**	**S**	**P**	**K**	**L**	**Q**	**A**	**N**	**L**	**K**
HEV-6	**X**	**X**	**X**	**D**	**X**	**V**	**D**	**A**	**P**	**P**						**A**	**A**	**X**	**X**										**T**	**X**	**X**	**X**	**X**	**X**	**I**	**X**	**X**	**X**	**P**	**X**	**X**	**X**	**M**	**S**	**X**	**X**	**X**	**X**	**A**
HEV-7	**V**	**G**	**X**	**S**	**X**			**X**	**A**	**P**								**X**	**X**										**X**	**X**	**X**	**X**	**X**	**X**	**C**	**X**	**P**	**P**	**P**	**X**	**S**	**X**	**Q**	**X**	**X**	**X**	**Q**	**P**	**X**
HEV-8			**P**	**E**	**A**	**X**	**L**	**X**	**K**	**P**						**X**	**X**	**V**	**X**	**C**	**E**	**P**	**X**	**G**	**P**	**L**	**L**	**X**	**X**	**T**	**X**	**X**	**X**							**X**	**X**	**G**	**A**	**P**	**T**	**E**	**A**	**X**	**X**

**Table:** (**b**)

						**ZNF787^(1)^ (3f)** **GATM^(1)^ (3f)** **RNA18S^(1)^ (3f)**										**ITIH2^(1)^ (3f)**											**KIF1B (3f)**				**PPR (3a, 3f, 3, 3j)**					**PPR (3e^(1)^, 3c, 3f^(1)^)** **PPR (3f-long)** **TRANSPEPTIDASE, SUBSTRATE BINDING DOMAIN AND SYNTHASE (3f)**												
											**8**								**9**											**10**		**11**			**12**				1AA 4c, 4d, 4a					1AA (2a)				
																																							**13**					**14**				
	82	83	84	85	86	87	88	89	90	91	92	93	94	95	96	97	98	99	100	101	102	103	104	105	106	107	108	109	110	111	112	113	114	115	116	117	118	119	120	121	122	123	124	125	126	127	128	129
HEV-1	**P**	**P**				**S**	**A**	**P**	**A**							**P**	**D**	**E**	**P**									**A**	**S**	**G**	**T**	**T**	**A**	**G**	**A**	**P**	**A**				**I**	**T**	**H**	**Q**	**T**	**A**	**R**	**H**
HEV-2	**L**	**P**				**D**	**V**	**T**	**D**							**G**	**S**	**R**	**P**									**S**	**G**	**A**	**R**	**P**	**A**	**G**		**P**	**N**			**P**	**N**	**G**	**V**	**P**				
HEV-3	**P**	**P**				**V**	**T**	**P**	**V**				**S**	**K**		**P**	**A**	**N**	**P**									**P**	**S**	**P**	**T**	**T**	**P**	**R**	**P**	**P**	**V**	**R**	**K**	**P**	**P**	**T**	**P**	**P**	**P**	**A**	**R**	**N**
HEV-4	**P**	**P**	**D**		**L**	**V**	**D**	**G**	**G**				**A**	**X**		**P**	**A**	**L**	**P**									**S**	**A**	**S**	**V**	**A**	**P**	**P**	**A**	**P**	**A**	**Q**		**P**	**V**	**X**	**P**	**S**	**G**	**P**	**R**	
HEV-5	**E**	**N**	**E**	**R**	**A**	**A**	**D**	**G**	**G**				**S**	**A**	**A**	**P**	**V**	**A**	**A**									**V**	**P**	**C**	**P**	**Q**	**P**			**P**	**A**	**Q**		**P**	**V**	**G**	**R**	**L**	**F**	**C**	**A**	**G**
HEV-6	**X**	**G**	**X**	**X**	**X**	**P**	**X**	**P**	**A**				**X**	**X**	**X**	**P**	**X**	**X**	**X**	**P**		**X**	**X**	**X**	**E**	**A**	**X**	**X**	**P**	**X**	**P**	**Q**	**X**			**X**	**X**	**X**	**S**	**X**	**A**	**X**	**X**	**X**	**X**	**A**	**X**	
HEV-7	**Q**				**X**	**P**	**X**	**P**							**X**	**X**	**X**	**X**	**X**	**P**	**X**	**X**	**P**	**X**	**X**	**X**	**X**	**S**	**X**		**X**	**X**				**P**	**A**	**Q**	**G**	**X**	**X**		**X**	**X**	**V**	**X**	**R**	**N**
HEV-8	**X**	**V**				**I**	**X**	**P**	**L**	**X**	**A**	**H**	**S**	**X**		**S**	**A**	**G**	**V**									**A**	**E**	**T**	**T**	**S**	**A**	**R**	**P**	**X**	**E**	**X**	**T**	**P**	**X**	**P**	**G**	**P**	**X**	**X**	**R**	**G**

**Table 4:** HEV-3 PPR duplications. HEV-3 consensus amino acid sequence and sequences with duplications are illustrated, along with 10 duplication blocks (A to J) and their sequences. The Table shows sequences that have a duplication, the sequence of this duplication and positions among which it is located. ^(a)^ RdRp insertion; ^(b)^ RRLL motif of C-terminal region; ^(c)^ L,D transpeptidase insertion; ^(d)^ Substrate-binding domain insertion; ^(e)^ Synthase insertion.

Blocks	A								B				C			D		E	F														G				H			I						J								
Positions	67	68	69	70	71	72	73	74	75	76	77	78	79	80	81	82	83	87	88	89	90	94	95	97	98	99	100	109	110	111	112	113	114	115	116	117	118	119	120	121	122	123	124	125	126	127	128	129						
HEV	I	W	V	L	P	P	P	S	E	G	S	A	I	D	P	P	P	V	T	P	V	S	K	P	A	N	P	P	S	P	T	T	P	R	P	P	V	R	K	P	P	T	P	P	P	A	R	N						
KC618402 KC618403								^74^	B	C	D	E	F	G	^a^	^75^																																						
MH184580 MH184581												^78^	C	D	E	F	G	B	^79^																																			
MF444088 MF444098 KJ917758												^78^	C	D	E	F	G	^79^																																				
*n* = 35																																^113^	I	^114^																				^113^
FJ956757																																^113^	G	^114^																				
EU495180																																^113^	G	H	I	F	^114^																	
MF444107																																^113^	G	H	I	J	F	^114^																
MF444036 MF444137																																^113^	G	H	I	J	F	^114^																
*n* = 6																																			^117^		H	I	^118^															
KJ917712																																			^117^		H	I	F	G	^118^													
MT899272																																			^117^		H	I	J	F	G	^118^												
*n* = 189																																			^117^		H	I	J	F	G	^118^												
KJ917720																																			^117^		H	I	J	^e^	F	^118^												
KJ917704																																			^117^		H	I	J	^b^	^d^	F	G	^118^										
MF444086																																			^117^		H	^c^	A	B	C	D	E	F	G	^118^								
MN646690 MN646691																																			^117^		H	I	J	^b^	A	B	C	D	E	F	G	^118^						
KJ917717																																			^117^		H	I	J	F	G	H	I	J	^b^	E	F	G	H	I	J	F	G	^118^

**Table 5:** Average of each AA category percentage comparing sequences with insertions, duplications, and none of them. NS: not significant.

Regulation Sites and AA Composition	Sequences with Human Fragment Insertions (*n* = 3)	Sequences with HEV Genome Duplication (*n* = 91)	Sequences without Insertions/DUPLICATIONS (*n* = 51)	*p* (Insertion/No Insertion)	*p* (Duplication/No Duplication)
Positively charged AA (%)	4.2	2.5	2.8	<0.05	NS
Negatively charged AA (%)	3.5	4.1	2.8	NS	<0.05
Polar AA (%)	3.6	3.2	4.3	NS	<0.05
Hydrophobic AA (%)	6.8	7.6	7.1	<0.05	<0.05
Aromatic AA (%)	0.99	0.8	1.0	<0.05	<0.05

## Supplementary Materials

The following are available online at https://www.mdpi.com/2076-2607/8/9/1417/s1, Table S1: HEV sequences with genomic rearrangements. Supplementary material 1: list of GenBank numbers of sequences included in the analysis. Figure S1. HEV PPR molecular phylogenetic analysis by Maximum Likelihood method, 1000Bt.
