# Supplementary material for: Proline-Rich Hypervariable Region of Hepatitis E Virus: Arranging the Disorder
Source: Microorganisms. 2020 Sep 15;8(9):1417. doi: 10.3390/microorganisms8091417 (PMC7564002; doi:10.3390/microorganisms8091417)
Supplement: Supplementary file 1 [file microorganisms-08-01417-s001.zip › Supplementary Table 1.docx]

**Supplementary Table 1.** HEV sequences with genomic rearrangements (insertions, duplications and deletions).

| **GB Number** | **Subtype** | **Genomic rearrangement** | **AA involved (n)** | **Location** | **Inserted fragment** | **Reference** |
| --- | --- | --- | --- | --- | --- | --- |
| KT591534 | 3f | Duplication | 93 | 1 | PPR | Lhomme 2020 |
| MK390971 | Unknown | Insertion | 1 | 2 | L |  |
| MF444043 | 3c | Insertion | 1 | 2 | P |  |
| 3 sequences CR-3 | 3f | Insertion | 28 | 3 | ApoC1 |  |
| MN646693 | 3f-long | Insertion | 36 | 4 | RPS17 | Lhomme 2020 |
|  |  | Duplication | 29 | 12 | PPR |  |
| MN646694 | 3m | Insertion | 50 | 5 | RPL6 | Lhomme 2020 |
|  |  | Deletion | 2 | 63, 67-68 |  |  |
| JN564006 | 3a | Insertion | 39 | 5 | RPS19 | Lhomme 2020 |
|  |  | Duplication | 3 | 10 | PPR |  |
| MF444145 | 3h | Insertion | 52 | 5 | RNF19A | Lhomme 2020 |
| HQ709170 | 3a | Insertion | 57 | 6 | RPS17 | Lhomme 2020 |
|  |  | Duplication | 5 | 10 | PPR |  |
| HQ389544, HW532736, JC087002, JQ679013 | 3a | Insertion | 57 | 6 | RPS17 |  |
| KC618403, KC618402* | 3c | Duplication | 62 | 6 | PPR | Lhomme 2020 |
| MH184580 | 3e | Duplication | 26 | 7 | PPR |  |
| MH184581 | 3e | Duplication | 28 |  |  |  |
| MF444088, MF444098, KJ917758 | 3f | Duplication | 29 | 7 | PPR |  |
| MN646692 | 3f-long | Insertion | 19 | 7 | EEF1a1P13 | Lhomme 2020 |
|  |  | Duplication | 29 | 12 | PPR |  |
|  |  | Deletion | 1 | 50 |  |  |
| MF444119 | 3f | Insertion | 75 | 8 | ZNF787 | Lhomme 2020 |
|  |  | Deletion | 1 | 57 |  |  |
| MN646689 | 3f-long | Insertion | 31 | 8 | GATM | Lhomme 2020 |
|  |  | Duplication | 29 | 12 | PPR |  |
|  |  | Deletion | 10 | 54, 94-111 |  |  |
| MN646695 | 3f-long | Insertion | 35 | 8 | RNA18S | Lhomme 2020 |
|  |  | Duplication | 29 | 12 | PPR |  |
|  |  | Deletion | 10 | 54, 94-111 |  |  |
| MN646696 | 3f-long | Insertion | 28 | 8 | Unknown |  |
|  |  | Duplication | 29 | 12 | PPR |  |
|  |  | Deletion | 10 | 54, 94-111 |  |  |
| KC166952*, KC166971 | 3f | Insertion | 25 | 9 | ITIH2 | Lhomme 2020 |
| MF444083 | 3f | Insertion | 53 | 10 | KIF1B | Lhomme 2020 |
|  |  | Deletion | 1 | 56 |  |  |
| KT727028 | 3a | Duplication | 7 | 11 | PPR |  |
| AB074918, AB074920, AB089824, AB481228, AB630970, KJ507955 | 3a | Duplication | 6 | 11 | PPR |  |
| AB591734, AF060668, HQ38543, AX181807, AX181808, BD378054, BD378055, KF303502, JQ679014 | 3a | Duplication | 4 | 11 | PPR |  |
| AF082843, AF060669, AX181882, AX181883, AY575857, AY575858, AY575859, BD378126, BD378127, FJ426403, FJ426404, AF082843, HQ389544, HW532736, JC087002, JQ679013, JN837481, KT447526, KT447528, MG833836 | 3a | Duplication | 5 | 11 | PPR |  |
| HW532737, JC087006 | 3a | Duplication | 3 | 11 | PPR |  |
| FJ956757 | 3f | Duplication | 4 | 11 | PPR |  |
| EU495180 | Unknown | Duplication | 23 | 11 | PPR |  |
|  |  | Deletion | 1 | 118 |  |  |
| MF444036, MF444137 | 3f | Duplication | 29 | 11 | PPR |  |
| MF444107 |  | Duplication | 20 |  |  |  |
| AY115488 | 3j | Duplication | 5 | 11 | PPR |  |
|  |  | Deletion | 1 | 40 |  |  |
| AB248520, AB291958, AB780450, AB780451, AB780452, AB780453 | 3e | Duplication | 13 | 12 | PPR |  |
| MF444086 | 3e | Duplication | 39 | 12 | PPR |  |
|  |  | Insertion | 10 |  | Transpeptidase |  |
| MN646690 | 3e | Duplication | 51 | 12 | PPR | Lhomme 2020 |
| MN646691 |  | Duplication | 52 |  | PPR |  |
| 136614HM56 | 3c | Duplication | 29 | 12 | PPR |  |
| KJ917704 | 3f | Duplication | 31 | 12 | PPR |  |
|  |  | Insertion | 13 |  | substrate-binding domain |  |
| KJ917717 |  | Duplication | 89 | 12 | PPR | Lhomme 2020 |
| KJ917720 | 3f | Insertion | 45 | 12 | Synthase |  |
|  |  | Duplication | 23 |  | PPR |  |
| KJ917712 | 3f-long | Duplication | 23 | 12 | PPR |  |
| MF444073 | 3f-long | Duplication | 25 | 12 | PPR |  |
|  |  | Deletion | 2 | 40, 117 |  |  |
| 140641HO47, 142571HAS51, MF444132 | 3f-long | Duplication | 28 | 12 | PPR |  |
| MF444057, MF444061, MF444140 | 3f-long | Duplication | 27 | 12 | PPR |  |
| KJ917766, KJ917678, KJ917689, KJ917691, KJ917714, KJ917671, KJ917716, KJ917741, 166285HSE75, 171921HM52, KJ917677, KJ917682, KJ917683, KJ917688, KJ917693, KJ917696, KJ917722, EU495171, KJ917726, KJ917743, KJ917752, KJ917756, 152434HNA47, 140053HM37, 140282HM32, 140572HM42, 141793MM51, 144223MBA70, 144461HM77, 144811MPO55, 144841HM39, 148523HGU53, 149221HM81, 149321MM55, 157033HM45, 159454HSE66, 160604MM45, 160981MSE55, 161913MNA48, 162025HBI52, 162065HM34, 163093HSE37, 163762HS61, 163913HS61, 165054HBI84, 165063HMLL56, 165463DMA60, 165604HA38, 165932HMA55, 167004HPO40, 168683HS50, 169675MLR60, 170115HBI50, 170261HPO51, 172285HBI52, 172573HM71, 172881HPO51, 173316HM61, 174184HM65, 174564MM54, 174866HM60, 175580HBI50, 176012HM59, 176031HGU39, 176473HM46, 176825HA75, 176964MM62, 177441HO52, 178134HOU53, 178844HBA48, 179031HPO51, 179333HM18, 179771MVA47, 180003HSE65, 180231HNA60, 180394HBI61, 181001HTO65, 181283HNA50, 181584HMA42, 181634MM53, 181774HB83, 181884HB62, 182094HSE57, 182344MCC36, 182714HSE54, 184161MMU79, 184774HM57, 185222MM44, 186143HM37, 186144HM56, 187041HPO51, 187723HM64, 188031HPO78, 188471HBI60, 189093HO69, 189150HM71, 189574HNA75, 189660HA56, 189674HCR38, 189821HNA76, 189874HB29, AB850879, EU495148, EU723514, EU723515, EU723516, KC166952, KC166971, H_Swine_148, H_Swine_151, H_Swine_363, H_Swine_54, Hi_Swine_206, Hi_Swine_49, JN906974, JN906975, JN906976, KC166967, KC166968, KC166969, KC166970, LC055972, LC055973, LC164712, MF444027, MF444034, MF444035, MF444038, MF444039, MF444040, MF444041, MF444045, MF444047, MF444048, MF444050, MF444051, MF444054, MF444055, MF444058, MF444059, MF444066, KT581448, MF444068, MF444069, MF444070, MF444076, MF444078, MF444081, MF444082, MF444084, MF444087, MF444090, MF444092, MF444093, MF444094, MF444095, MF444096, MF444097, MF444100, MF444103, MF444105, MF444108, MF444112, MF444117, MF444123, MF444124, MF444125, MF444127, MF444129, MF444130, MF444133, MF444134, MF444138, MF444139, MF444142, 174953HGC54 | 3f-long | Duplication | 29 | 12 | PPR |  |
| KT591533 | 3f | Deletion | 1 | 40 |  |  |
| KC166969 |  | Deletion | 2 | 40, 120 |  |  |
| KJ917673 | Unknown | Deletion | 2 | 49, 126 |  |  |
| 189874HB29, MF444101 | 3f | Deletion | 1 | 49 |  |  |
| AB740221 | 3ra | Deletion | 15 | 50, 52, 53, 82-90, 94-99, 112 |  |  |
| AB740220, AB740222, FJ906895, FJ906896, GU937805, JQ768461, JX109834, JX565469, KX227751, KY436898, KY496200, LC484431, MF444099, MF480297, MF480298 |  | Deletion | 13 | 52, 82-90, 94-99, 112 |  |  |
| AF455784 | 3g | Deletion | 9 | 37, 40-41, 50-52, 63, 81-82 |  |  |
| MK390971 | 3 | Insertion | 1 | 2 | Leucine |  |
| MF444043 | 3c | Insertion | 1 | 2 | Proline |  |
|  |  | Deletion | 1 | 55 |  |  |
| MF444108 | 3f | Deletion | 1 | 53 |  |  |
| MF444128 | 3c | Deletion | 2 | 53, 63, 68 |  |  |
| MF444115 |  | Deletion |  | 53-54 |  |  |
| MF444141 | 3e | Deletion | 1 | 56 |  |  |
| MF444116 | 3f | Deletion | 3 | 56-57, 76 |  |  |
| KJ917751 | Unknown | Deletion | 1 | 57 |  |  |
| MF444140 | 3f | Deletion | 3 | 64, 79, 99 |  |  |
| MF444103 |  | Deletion | 1 | 67 |  |  |
| MF444032 |  | Deletion | 5 | 68, 83, 87 121, 126 |  |  |
| MF444104 |  | Deletion | 2 | 68, 90 |  |  |
| MF444129 |  | Deletion | 1 | 68 |  |  |
| MF444034 |  | Deletion | 1 | 71 |  |  |
| KU176131 | 3h | Deletion | 1 | 63, 74 |  |  |
| KJ917762 | Unknown | Deletion | 2 | 63, 76, 115 |  |  |
| MF444131 | 3l | Deletion | 1 | 63, 77 |  |  |
| MF444049 | 3c | Deletion | 1 | 89 |  |  |
| MF444072 |  | Deletion |  | 90 |  |  |
| MF444057 | 3f | Deletion | 1 | 94 |  |  |
| MF444133 | 3f | Deletion | 1 | 97 |  |  |
| 186633MA46 |  |  |  |  |  |  |
| MH184579 | 3e | Deletion | 1 | 111 |  |  |
| AB481226 |  | Deletion |  | 112 |  |  |
| 175362HM68 | 3m | Deletion | 1 | 113 |  |  |
| MF444054 | 3f | Deletion | 1 | 114 |  |  |
| MF444135 |  |  |  |  |  |  |
| KJ917744 | Unknown | Deletion | 1 | 115 |  |  |
| MF444143 | 3c | Deletion | 1 | 115 |  |  |
| MF444061 | 3f | Deletion | 1 | 116 |  |  |
| MF959764 | 3 | Deletion | 1 | 119 |  |  |
| EU495185 | Unknown | Deletion |  | 121 |  |  |
| MF444052 | 3f | Deletion | 1 | 122 |  |  |
| MG833836 | 3a | Deletion | 1 | 124 |  |  |
| MF444130 | 3f | Deletion | 1 | 125 |  |  |
| KJ917737 | Unknown | Deletion | 1 | 126 |  |  |
| KX578717 | 2a | Insertion | 1 | 13 |  |  |
