# Supplementary material for: Proline-Rich Hypervariable Region of Hepatitis E Virus: Arranging the Disorder
Source: Microorganisms. 2020 Sep 15;8(9):1417. doi: 10.3390/microorganisms8091417 (PMC7564002; doi:10.3390/microorganisms8091417)
Supplement: Supplementary file 1 [file microorganisms-08-01417-s001.zip › Supplementary Figure 1.docx]

**Supplementary Figure 1.** HEV PPR molecular phylogenetic analysis by Maximum Likelihood method, 1000Bt.
