# Supplementary material for: Proline-Rich Hypervariable Region of Hepatitis E Virus: Arranging the Disorder
Source: Microorganisms. 2020 Sep 15;8(9):1417. doi: 10.3390/microorganisms8091417 (PMC7564002; doi:10.3390/microorganisms8091417)
Supplement: Supplementary file 1 [file microorganisms-08-01417-s001.zip › Supplementary material 1.docx]

GenBank accession number: **Smith et al.** ([25](#_ENREF_25)) sequences: M73218 (HEV-1a), L08816 (HEV-1b), X98292 (HEV-1c), AY230202 (HEV-1d), AY204877 (HEV-1e), JF443721 (HEV-1f), LC225387 (HEV-1g), FJ457024 (HEV-1), MH918640 (HEV-1), KX578717 (HEV-2a), MH809516 (HEV-2b-provisional), AF082843 (HEV-3a), AP003430 (HEV-3b), FJ705359 (HEV-3c), AB248521 (HEV-3e), AB369687 (HEV-3f), AF455784 (HEV-3g), JQ013794 (HEV-3h), FJ998008 (HEV-3i), AY115488 (HEV-3j), FJ906895 (HEV-3ra), AB369689 (HEV-3k), JQ953664 (HEV-3l), KU513561 (HEV-3m), AB290313 (HEV-3Mongolia), MF959765 (HEV-3Italy), LC260517 (HEV-3Japan), MK390971 (HEV-3Italy), MF959764 (HEV-3Italy), KP294371 (HEV-3Germany), 4a AB197673 (HEV-4a), DQ279091 (HEV-4b), AB074915 (HEV-4c), AJ272108 (HEV-4d), AY723745 (HEV-4e), AB220974 (HEV-4f), AB108537 (HEV-4g), GU119961 (HEV-4h), AB369690 (HEV-4i), MK410048 (HEV-4China), AB369688 (HEV-4Japan), AB573435 (HEV-5a), AB602441 (HEV-6a), AB856243 (HEV-6Japan), KJ496143 (HEV-7a), KJ496144 (HEV-7UAE), KX387865 (HEV-8a) and MH410174 (HEV-8China). **Genotype 1** sequences: AF051830, AF076239, AF459438, DQ459342, JF443717, JF443718, JF443719, JF443720, LC061267, MH504157, AF444002, AF444003, D11092, D11093, JQ655734, M94177, NC001434, JF443717, AB720034, AB720035, JF443722, JF443723, JF443724, JF443725, JF443726, MH074880, MH504154, MH504162, MH991993, MH991994, MH991995, MH991996, MH991997, MH991998, MH991999, MH992000, MH992001, MH992002, MH992003, MH992004, MH992005, MH992006, MH992007, MH992008, MH992009, MH992010, MH992011, MH992012, MH992013, LC314155, LC314156, LC314157, LC314158, MH504155, MH504156, MH504158, MH504159, MH504160, MH504161, MH504163 and MN401238. **Genotype 3** sequences: AB073912, AB222184, KT633715, EU495181, EU495184, EU495185, FJ527832, KJ507956, KT727028, AB074918, AB074920, AB089824, AB481228, AB630970, KJ507955, AB591734, AF060668, HQ389543, AX181807, AX181808, BD378054, BD378055, KF303502, JQ679014, AF082843, AF060669, AX181882, AX181883, AY575857, AY575858, AY575859, BD378126, BD378127, FJ426403, FJ426404, HQ389544, HQ709170, HW532736, JC087002, JQ679013, JN837481, KT447526, KT447528, MG833836, JN564006, HW532737, JC087006, AB091394, AB189070, AB189071, AB189072, AB189073, AB189074, AB189075, AB222182, AB222183, AB236320, AB246676, AB291951, AB291952, AB291953, AB291954, AB291955, AB291956, AB291957, AB291960, AB291962, AB291963, AB301710, AB362839, AB362840, AB362841, AB362842, AB362843, AB369691, AB425830, AB425831, AB437316, AB437317, AB437318, AB437319, AB443623, AB443624, AB443625, AB443626, AB443627, AB481229, AB591733, AB593690, AB630971, AB698071, LC126331, LC126332, LC386855, LC439299, AB290312, JQ013794, KC618402, KC618403, KT159771, KU176129, KX172133, MF444042, MF444043, MF444049, MF444065, MF444072, MF444085, MF444106, MF444111, MF444114, MF444115, MF444122, MF444128, MF444143, MG783569, MG783570, AB248520, AB291958, AB780450, AB780451, AB780452, AB780453, MF444086, MN646690, MN646691, AB481226, AB248521, AB248522, FJ998015, HM055578, JQ013795, JQ026407, JQ953665, KF922359, KP698919, MF444109, MF444141, MH184579, MH184580, MH184581, MH184582, MH184583, MH184584, AB291961, EU360977, EU375463, EU723512, EU723513, FJ653660, JQ013795, JQ953666, KT591534, MF444083, KT447527, KT581444, KT591532, KT591533, KY232312, KY232313, MF444028, MF444029, MF444032, MF444046, MF444052, MF444067, MF444079, MF444088, MF444098, MF444101, MF444102, MF444104, MF444113, MF444116, EU360977, KT581443, KT581445, KT581446, KT581447, KU747141, KU747142, KU980235, MF444091, MF444053, MF444075, MF444135, MF444119, FJ956757, EU495180, MF444036, MF444137, MF444107, MN646689, MN646695, MN646696, MN646692, MN646693, KJ917704, KJ917717, KJ917720, EU495171, AB850879, EU495148, EU723514, EU723515, EU723516, KC166952, KC166971, JN906974, JN906975, JN906976, KC166967, KC166968, KC166969, KC166970, LC055972, LC055973, LC164712, MF444027, MF444034, MF444035, MF444038, MF444039, MF444040, MF444041, MF444045, MF444047, MF444048, MF444050, MF444051, MF444054, MF444055, MF444057, MF444058, MF444059, MF444061, MF444066, KT581448, MF444068, MF444069, MF444070, MF444073, MF444076, MF444078, MF444081, MF444082, MF444084, MF444087, MF444090, MF444092, MF444093, MF444094, MF444095, MF444096, MF444097, MF444100, MF444103, MF444105, MF444108, MF444112, MF444117, MF444123, MF444124, MF444125, MF444127, MF444129, MF444130, MF444132, MF444133, MF444134, MF444138, MF444139, MF444140, MF444142, KU176131, KU176132, KY780957, MF346773, MF444037, MF444056, MF444077, MF444110, MF444120, MF444136, MF444145, MG573193, AB290312, MF346772, KP294371, AB740232, LC131066, LC176492, LC176493, KY766999, MF444131, MG674164, MF444030, MF444089, MN646694, AB740220, AB740221, AB740222, FJ906896, GU937805, JQ768461, JX109834, JX565469, KX227751, KY436898, KY496200, LC484431, MF444099, MF480297, MF480298, KJ917667, KJ917768, KJ917771, KJ917772, KJ917773, KJ917774, KJ917669, KJ917699, KJ917700, KJ917702, KJ917707, KJ917708, KJ917710, KJ917724, KJ917727, KJ917728, KJ917730, KJ917732, KJ917734, KJ917673, KJ917736, KJ917737, KJ917738, KJ917739, KJ917744, KJ917746, KJ917747, KJ917749, KJ917751, KJ917754, KJ917759, KJ917761, KJ917762, KJ917764, KJ917766, KJ917678, KJ917689, KJ917691, KJ917714, KJ917671, KJ917716, KJ917741, KJ917677, KJ917682, KJ917683, KJ917688, KJ917693, KJ917696, KJ917712, KJ917722, KJ917726, KJ917743, KJ917752, KJ917756 and KJ917758. **Genotype 4** sequences: AB161719, AY621103, AB197674, EF077630, EU366959, FJ763142, GU119960, GU119961, HQ634346, JQ655733, KC492825, KC692453, LC037955, MK410045, MK410046, MK410047, MK410049, MK410050, MK410051, MK410052, MK410053, AB253420, AB291964, EU676172, JX85579, LC0422320, LC428039, LC436449, LC436450, AB080575, AB091395, AB097811, AB097812, AB099347, AB193176, AB193177, AB193178, AB200239, AB220971, AB220972, AB074917, AB220973, AB220975, AB220976, AB220977, AB220978, AB220979, AB291959, AB291965, AB291966, AB291967, AB291968, AB481227, LC022745, LC387631, AY594199, FJ610232, GU206559, GU361892, JQ655736, KC163335, KF176351, KX531115, KX827238, MK41004, AB698654, LC387632, JQ655735, JQ740781, KF736234, KJ155502, KM253769, KR872414, KR872415, KR872416, KR872417, KU356182, KU356183, KU35618, KU356185, KU356186, KU356187, KU356188, KU356189, AB521805, AB521806, AB602439, AB602440, AB909124, AB909125, DQ450072, EF570133, HM439284, JF915746 and JQ993308. **Genotype 8** sequences: MH410175, MH410176, KX387865 and KX387866
